# RaDiCo, the French national research program on rare disease cohorts

**DOI:** 10.1186/s13023-021-02089-5

**Published:** 2021-10-29

**Authors:** Serge Amselem, Sonia Gueguen, Jérôme Weinbach, Annick Clement, Paul Landais, for the RaDiCo Program

**Affiliations:** 1 RaDiCo, Inserm, Trousseau Hospital, Paris, France; 2 Sorbonne Université, Inserm U933, Childhood Genetic Disorders, Trousseau Hospital, 26 rue du Dr. Arnold Netter, 75012 Paris, France; 3 Department of Paediatric Respiratory Medicine, Trousseau Hospital, Assistance Publique Hôpitaux de Paris, Paris, France; 4 EA2415, University Clinical Research Institute, Montpellier University, Montpellier, France; 5 Present Address: Direction Générale de La Santé, Ministry of Health, Paris, France

**Keywords:** Rare diseases, e-Cohorts, Mutualized platform, Cloud computing, Infrastructure-as-a service, GDPR, Interoperability, Health Data Hub, European Reference Networks, Rare Disease European Joint Program

## Abstract

**Background:**

Rare diseases (RDs) affect nearly 3 million people in France and at least 26–30 million people in Europe. These diseases, which represent a major medical concern, are mainly of genetic origin, often chronic, progressive, degenerative, life threatening and disabling, accounting for more than one third of all deaths occurring during infancy. In this context, there are needs for coordinated information on RDs at national/international levels, based on high quality, interoperable and sharable data. The main objective of the RaDiCo (Rare Disease Cohorts) program, coordinated by Inserm, was the development of RD e-cohorts via a national platform. The cohort projects were selected through a national call in 2014. The e-cohorts are supported by an interoperable platform, equivalent to an infrastructure, constructed on the "cloud computing" principle and in compliance with the European General Data Protection Regulation. It is dedicated to allow a continuous monitoring of data quality and consistency, in line with the French Health Data Hub.

**Results:**

Depending on cohorts, the objectives are to describe the natural history of the studied RD(s), identify the underlying disease genes, establish phenotype-genotype correlations, decipher their pathophysiology, assess their societal and medico-economic impact, and/or identify patients eligible for new therapeutic approaches. Inclusion of prevalent and incident cases started at the end of 2016. As of April 2021, 5558 patients have been included within 13 RD e-cohorts covering 67 diseases integrated in 10 European Reference Networks and contributing to the European Joint Program on RDs. Several original results have been obtained in relation with the secondary objectives of the RaDiCo cohorts. They deal with discovery of new disease genes, assessment of treatment management, deciphering the underlying pathophysiological mechanisms, diagnostic approaches, genotype–phenotype relationships, development and validation of questionnaires relative to disease burden, or methodological aspects.

**Conclusion:**

RaDiCo currently hosts 13 RD e-cohorts on a sharable and interoperable platform constructed on the “cloud computing” principle. New RD e-cohorts at the European and international levels are targeted.

## Background

Rare Diseases (RDs) represent a major health care issue. A disease is called rare in Europe when it affects fewer than 5 in 10,000 persons. Depending on the definition used, it is estimated that there are 7000 to 8000 RDs. Taken as a whole, RDs affect at least 26–30 million people in Europe. These diseases involve children in about 75% of cases. They are often chronic, progressive, degenerative, life threatening and disabling, accounting for more than one third of all deaths occurring during infancy. A large number of these diseases lead to a significant decrease of life expectancy and most of them have a significant impact on patients’ quality of life and health care systems. This is a major medical concern since, for most of these diseases, there is no available cure. RDs are mainly of genetic origin. At the date of writing this article, 4425 genes underlying 6868 disease phenotypes have been discovered (https://www.omim.org/statistics/geneMap), but the genes underlying a number of known Mendelian phenotypes are still unknown, and additional Mendelian conditions have yet to be recognized. Thus, there are needs for high quality, interoperable and sustainable creation and monitoring of (inter)national cohorts of patients with rare diseases.

Coordinated care and research on RDs appear critical for patients and families, in order to better describe the natural history of the disease, to improve the diagnostic procedures, to decipher the underlying pathophysiological mechanisms and to better stratify patients for targeted clinical trials and treatments through personalized medical approaches. It is also expected that research on RDs will increase our understanding of the pathophysiology of common chronic diseases, as RDs often represent a “model of dysfunction” severely affecting a limited number of biological pathways.

Identifying the locks is essential. Yet, RD clinical management and research in France and Europe have been hampered by a lack of resources at several levels: few scientists work on only one given disease; few patients per disease and patients are scattered over large geographic areas, causing difficulties in gathering data on their disease; existing databases as well as biological collections, when existing, are usually local, small, incomplete, not always quality-controlled, of heterogeneous formats and contents, and are rarely accessible or standardized for allowing interoperability; phenomes are often complex and partially described along with time, with insufficient interdisciplinary cooperation.

The need to promote networks of expertise for RDs in order to improve both RD clinical care and research has been considered a priority in France since 1995. Indeed, RDs occupy an important place in public health in France: Orphanet, an informational RD website and a directory of expert services was launched in 1997 [1] by the Health Ministry; the French federation of patients’ organizations (“Alliance Maladies Rares”), was launched in February 2000 [2]; and the RD Scientific Interest Group was created to fund research in the field of RDs. The creation of the first French National Rare Disease Plan (PNMR1, 2005–2008) [3] allowed access to high quality care, and treatment was facilitated by the creation, at the national level, of RD Reference Centers and Competence Centers. The second Plan (PNMR2, 2011–2016) consolidated previous achievements, aiming at reinforcing national and international cooperation [4]. Twenty University genetic laboratories were equipped with the Next Generation Sequencing technology for clinical use. In 2014, RD Reference and Competence Centers were grouped into 23 thematic RD Healthcare Networks (RDHNs) (https://solidarites-sante.gouv.fr/soins-et-maladies/prises-en-charge-specialisees/maladies-rares/article/l-offre-de-soins). This move anticipated the 23 RD European Reference Networks (ERNs) that were launched at the end of 2016 (https://ec.europa.eu/health/ern_en). The RDHNs coordinate diagnosis, provision of health, social care and training; they collect healthcare data, develop research programs and write National Protocols for Diagnosis and Care.

One main objective of the 3^rd^ RD Plan (PNMR3, (2018–2022) [5], in line with the International Rare Diseases Research Consortium [6], was to provide an accurate diagnosis within a year of the first specialty medical consultation. The PNMR3, created 109 RD Coordinating Centers (RDCC) associated to 386 constitutive centers and 1840 competence centers, and reinforced the links with European research initiatives on RDs. It also aims at strengthening the role of RaDiCo in integrating RDs research data (PNMR3, Action 11.4).

A need for launching RD cohorts. In this fast-mutating context, RD professionals highlighted the critical need to implement nation-wide, multidisciplinary, high-quality cohort studies in order to address key scientific and medico-economic questions. In this context, the request was high to get access to appropriate resources, methods and tools for collecting RD data at the (inter)national level. Given the specificities of RDs—limited number of patients per country, scarcity of relevant knowledge and expertise, and fragmentation of research—they have been considered as a distinctive domain of very high national and European added value. A supporting research program was therefore required to provide essential information on disease history and characteristics, and to foster the identification of underlying molecular mechanisms, genotype/phenotype correlations. New knowledge would ultimately lead to better targeted care and treatments. The structuring boost given by the PNMR2 was considered as an opportunity to make collective efforts for building RD cohorts and therefore to propose the RaDiCo (Rare Disease Cohorts) project to the national call on cohorts of the first Investments for the Future Program (https://anr.fr/en/investments-for-the-future/investments-for-the-future/) launched by the French Ministry of Research in 2010 [7].

The objective of the RaDiCo project was twofold. On one hand, the scientific objective was to set up several RD e-cohorts with the following aims according to the idiosyncrasy of each cohort: (i) Describe the natural history of the targeted RDs; (ii) Identify the disease-causing genes; (iii) Establish genotype–phenotype correlations; (iv) Decipher the underlying pathophysiological mechanisms; (v) Identify new therapeutic avenues; (vi) Estimate their societal and medico-economic impact; (vii) Identify patients eligible for new therapeutic approaches; (viii) Define a methodological strategy of analysis for cohorts which recruit both prevalent and incident cases, that require adapted modeling and bias analyses. In a rare disease setting, cases are either prevalent (already known with a past history) or incident (recently discovered). Most patients are prevalent since diseases by definition are rare. Then the analysis of such observational data has to take into account prospective as well as retrospective information. As already outlined, retrospective data are more bias-sensitive than prospective data and this must be considered in the analysis (https://www.strobe-statement.org). On the other hand, to reach these scientific objectives, another goal was to build a national operational platform, equivalent to an Infrastructure as a Service, for implementing a potentially unlimited number of e-cohorts consisting of prevalent and incident cases.

**Fig. 1:**
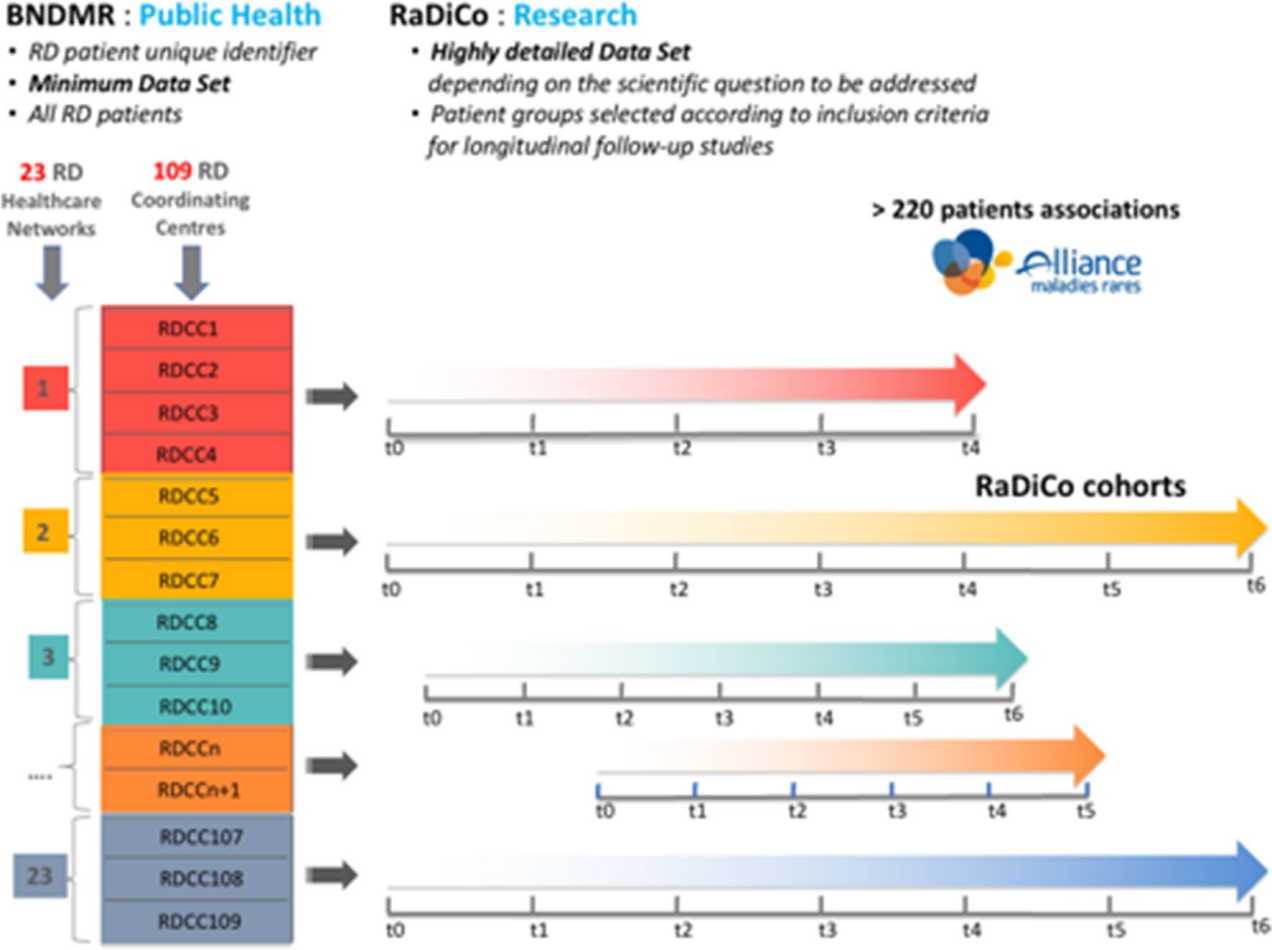
French RD Healthcare Networks, BNDMR (RD National Databank) and RaDiCo

Such cohort projects had to be closely articulated with the above-mentioned established networks on RDs. The PNMR2 also fostered the development of the National RD Data Bank (“*Banque Nationale de Données Maladies Rares*” (BNDMR), Fig. 1) built as part of the CEMARA initiative [8], which started in 2007 at Necker Enfants-Malades Hospital (AP-HP).The BNDMR, which is dedicated to public health issues, aims at collecting general epidemiological and public health data on all patients with a RD in France, on the basis of a common RD minimum data set [9] and a unique identifier [10] for each RD patient.

The RD cohorts also had to integrate, whenever appropriate, non-French RD expert centers and patients, in order to overcome the low number of patients in one single country with respect to the sample sizes needed for proper statistical power, thereby anticipating the emergence of the future RD European Research Networks.

## Results

Thirty-three letters of intent were received after the publication of the RaDiCo call for RD cohorts (see Material and Methods). This call led to select 16 national and/or European RD cohort projects on July 15th, 2014. Among these, 3 have been discontinued, secondary to decisions of the Scientific and Plenary committees of the RaDiCo program (see Material and Methods): after demand from the principal investigators (PIs) for two of them, since the cohorts had not started, and another in 2019 because it could not start in due time. The groups of RDs targeted by the 13 current cohorts appear in Table 1.

**Table 1:** The 13 ongoing RaDiCo e-cohorts (acronyms in alphabetic order)

Cohorts’ acronyms	Diseases	RDHN^a^	Corresponding ERN^b^
AC-ŒIL	Congenital eye defects	SENSGENE AnDDI-Rares	ERN-Eye
AcoStill	Still’s disease	FAi2R	ERN-Rita
COLPAC	LPAC syndrome	FILFOIE	RARE-LIVER
DCP	Primary ciliary dyskinesia	RESPIFIL	ERN-Lung
ECYSCO	Cystinosis	ORKiD	ERKnet
EURBIO-Alport	Alport syndrome	ORKiD	ERKnet
FARD	Rare skin diseases burden	FIMARAD	ERN Skin
GenIDA	Intellectual deficiency and Autism spectrum disorders	DéfiScience AnDDi-Rares	ERN ITHACA
IDMet	Imprinting disorders	FIRENDO OSCAR DéfiScience	ENDO-ERN
MPS	Mucopolysaccharidoses	G2M	MetabERN
PID	Idiopathic interstitial pneumonia	RESPIFIL	ERN-Lung
PP	Periodic paralysis	FILNEMUS	ERN euro-NMD
SEDVasc	Vascular Ehlers-Danlos syndrome	FAVA-Multi	VASCERN

^a^RDNH: Rare Disease Healthcare Network

^b^ERN: European Research Network

**Fig. 2:**
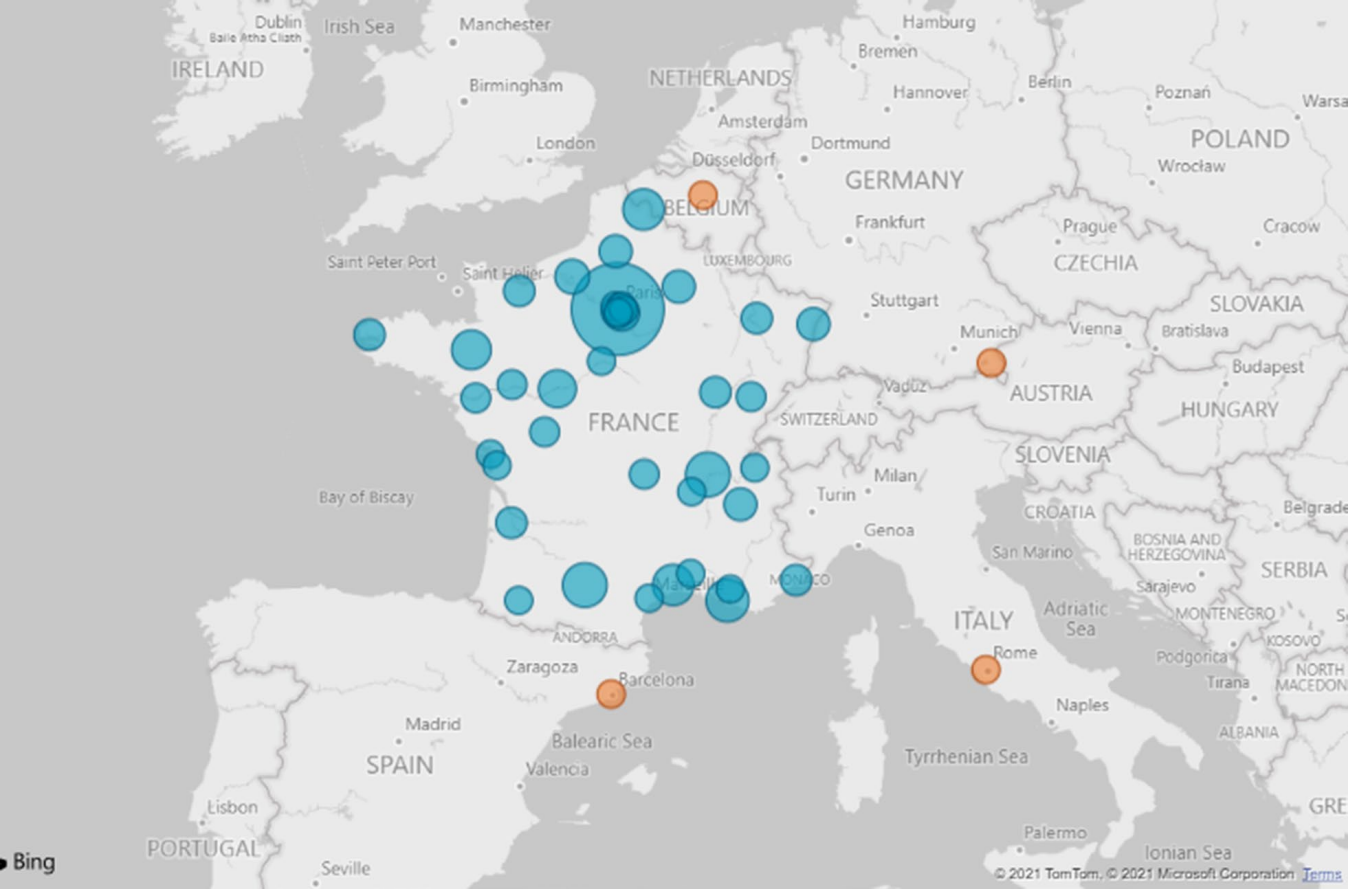
Geographical distribution of RDs Coordinating Centres contributing to RaDiCo e-cohorts (France (blue); other European countries (orange)

**Fig. 3:**
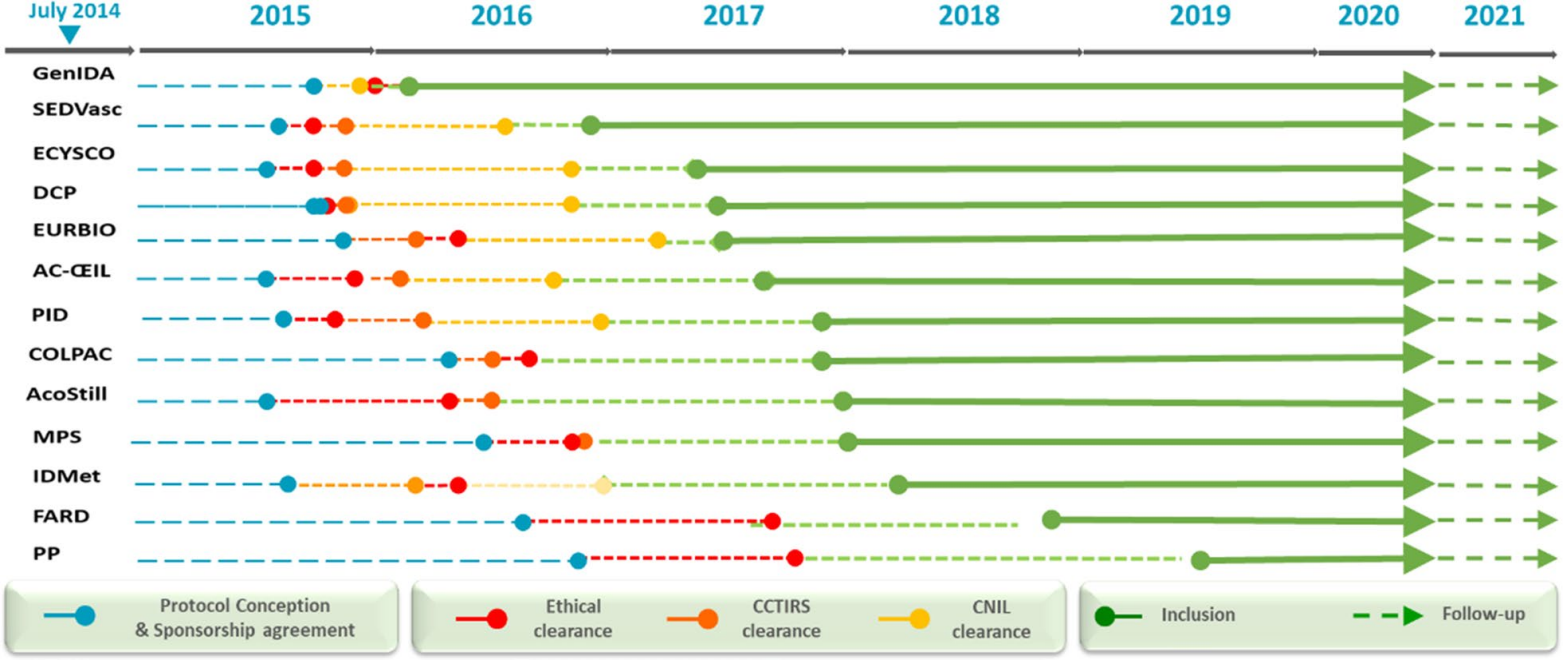
RaDiCo cohorts' preparatory work and 2014–2021 follow-up (CCTIRS, «Comité Consultatif pour le Traitement de l'Information en Recherche en Santé»; CNIL «Commission Nationale Informatique et Libertés»)

The general framework of the RaDiCo cohorts is presented below (Table 2), with or without associated biocollections at each site, together with the planned inclusion and follow-up period. The start of the inclusions and the inclusion targets are mentioned. All cohorts are multicentric, mainly national but also European (SEDVasc and ECYSCO cohorts, see Table 2) and international (GenIDA). For all the cohorts, a total of 11,650 included patients are expected at the end of the inclusion period (July 2027). As of April 2021, 5558 patients had been included into 13 RD e-cohorts, covering 67 diseases from ~ 300 affiliated expert centers (Fig. 2). Each implementation step of the e-cohorts is presented in Fig. 3.

**Table 2:** RaDiCo cohorts: general framework

RaDiCo-Cohort Acronyms	Biocollections	Inclusion period (years)	Follow-up period (years)	Start of inclusions	Inclusion targets
AC-ŒIL	Diagnosis	10	10	Jul.-2017	800
AcoStill	Yes	5	5	Dec.-2017	500
COLPAC	Yes	3	5	Nov.-2017	550
DCP	Diagnosis	5	5	May-2017	700
ECYSCO	Diagnosis	2	2	Apr.-2017	400
EURBIO Alport	Yes	2	3	May-2017	1000
FARD	Yes	4	5	Mar.-2018	900
GenIDA	No	5	5	Nov.-2016	1000
IDMet	Yes	5	10	Mar.-2018	2000
MPS	Yes	2	5	Dec.-2017	1000
PID	Diagnosis	5	5	Nov.-2017	2400
PP	No	1	1.5	Jun.-2019	60
SEDVasc	Diagnosis	4	3	Dec.-2016	340
				Expected total inclusions	11,650

The electronic cohorts (e-cohorts) were supported by a platform on which RaDiCo’s work system was developed. Its activities was devoted to capturing, transmitting, storing, retrieving, manipulating and displaying information. This platform fulfills the necessary requirements of an Information System. It has been positively assessed by an independent external auditor. Moreover, it contributed to the design of the Information System of the Inserm “France Cohortes” program designed to support not only RD e-cohorts, but also cohorts of patients with common multifactorial disorders, as well as population-based epidemiological cohorts.

Primary objectives of the RD e-cohorts are not yet achieved. This situation mainly concerns the description of the natural history of the targeted RD(s). According to the study protocol of each cohort, this objective relies on the follow-up of each patient enrolled in a given cohort. Thus we have to wait for the data freeze procedure to be allowed to analyze the data collected in the purpose of exploring the primary endpoint. In contrast, this is not the case for the secondary objectives. They deal with discovery of new disease genes, assessment of treatment management, deciphering the pathophysiology and diagnostic approaches, genotype–phenotype relationships, development and validation of questionnaires relative to the diseases burden, or methodological aspects (Table 3).

**Table 3:** International publications so far associated to the RaDiCo program

Discovery of new disease genes	*TTC12* loss-of function mutations cause primary ciliary dyskinesia and unveil distinct dynein assembly in motile cilia vs. flagella [11] Lack of *GAS2L2* causes primary ciliary dyskinesia by impairing cilia orientation and mucociliary clearance [12] Mutations in outer dynein arm heavy chain *DNAH9* cause motile cilia defects and situs inversus [13] Mutations in *DNAJB13*, encoding an HSP40 family member, cause primary ciliary dyskinesia and male infertility [14] de novo missense variants in *FBXW11*, a gene that encodes an F-box protein involved in ubiquitination and proteosomal degradation [15]
Assessment of treatment management	Vascular Ehlers-Danlos syndrome – Long-term observational study [16]
Pathophysiology and diagnostic approaches	Accuracy of clinical diagnostic criteria for patients with vascular Ehlers-Danlos syndrome in a tertiary referral centre [17] Functional assessment and phenotypic heterogeneity of *SFTPA1* and *SFTPA2* mutations in interstitial lung diseases and lung cancer [18] Pulmonary fibrosis in children [19] Chronic interstitial lung diseases in children: diagnosis approaches [20] Pulmonary hemosiderosis in children with Down syndrome: a national experience [21] Paediatric sarcoidosis [22] Genetic causes and clinical management of pediatric interstitial lung diseases [23]
Genotype–phenotype relationships	Infertility in an adult cohort with primary ciliary dyskinesia: phenotype-gene association [24] Primary ciliary dyskinesia gene contribution in Tunisia: Identification of a major Mediterranean allele [25] Alport syndrome: a unified classification of genetic disorders of collagen IV α345 [26] Genetics of anophthalmia and microphthalmia. Part 1: Non-syndromic anophthalmia/microphthalmia [27]
Development and validation of burden questionnaires and Quality of life	Burden of albinism: development and validation of a burden assessment tool [28] Burden of adult neurofibromatosis 1: development and validation of a burden assessment tool [29] Health-related quality of life in infants and children with interstitial lung disease [30]
Methodological aspects	Federating patients identities: the case of rare diseases [10] Cerberus, an access control scheme for enforcing least privilege in patient cohort study platforms: [31] National registries of rare diseases in Europe: an overview of current situation and experiences [32] Recommendations for improving the quality of rare disease registries [33] Data quality in rare diseases registries [34]

Discovery of new disease genes—Four genes, i.e., *TTC12* [11], *GAS2L2* [12], *DNAH9* [13] and *DNAJB13* [14], whose mutations are responsible for Primary Ciliary Dyskinesia (PCD) have been identified through molecular and cellular studies performed in the framework of the RaDiCo-DCP cohort. This cohort has been built on the deep phenotyping of the patients, which includes the ultrastructural defects of the microtubule-based structure of motile cilia and sperm flagella (i.e., the ciliary and flagellar axonemes). These organelles contain several dynein arms, each of them consisting of multiprotein complexes that carry an ATPase activity required for ciliary/flagellar motility. Specific phenotypes have been associated with mutations in these new genes that code for different classes of proteins. TTC12 is believed to be a co-chaperone involved in the cytoplasmic pre-assembly of dynein arms [11]; the lack of GAS2L2 causes PCD by impairing cilia orientation and mucociliary clearance [12], whereas DNAH9 encodes one of the axonemal dynein chains [13]. As for *DNAJB13*, it encodes an HSP40 family member involved in the proper building of the ciliary and flagellar axoneme [14]. In addition, besides the identification of those new molecular causes of PCD, one of the key results obtained through functional studies performed on both patients’ primary airway epithelial cells (AECs) and CRISPR-Cas9-edited human primary AECs is the existence of distinct dynein assembly mechanisms in human motile cilia versus flagella [11]. As for the developmental eye defects reported in patients from the RaDiCo-AC-OEIL cohort, de novo missense variants have been identified in *FBXW11*, a gene that encodes an F-box protein involved in ubiquitination and proteosomal degradation [15].

Assessment of treatment management—From a therapeutic viewpoint, as shown in the RaDiCo-SEDVasc cohort, which is dedicated to patients with a rare genetic connective tissue disorder called vascular Ehlers-Danlos syndrome, the assessment of treatment management has revealed the impact of different therapies on morbidity and mortality. Indeed, in this disease condition due to mutations in *COL3A*, it has been shown that patients treated with celiprolol —a beta blocker— had a better survival than those not treated with celiprolol and that the observed reduction in mortality was dose-dependent [16].

Pathophysiology and diagnostic approaches—Several aspects were explored: Accuracy of clinical diagnostic criteria for patients with vascular Ehlers-Danlos syndrome in a tertiary reference center for the RaDiCo-SEDVasc cohort [17]; In the RaDiCo-PID cohort, functional assessment and phenotypic heterogeneity of *SFTPA1* and *SFTPA2* mutations in interstitial lung diseases and lung cancer [18]; Pulmonary fibrosis in children [19]; Diagnosis approaches of chronic interstitial lung diseases in children: [20–22]; or genetic causes and clinical management of pediatric interstitial lung diseases [23].

Genotype–phenotype relationships – For the RaDiCo-DCP cohort, infertility in an adult cohort with primary ciliary dyskinesia [24]; Identification of a major Mediterranean allele in primary ciliary dyskinesia gene [25]. For the RaDiCo-EURBIO Alport cohort, a unified classification of genetic disorders of collagen IV α345 in Alport syndrome [26]. For the RaDiCo AC-OEIL cohort, review of the genetics of non-syndromic anophthalmia/microphthalmia [27].

Development and validation of burden questionnaires and quality of life study—Two questionnaires were validated in order to support the RaDiCo–FARD cohort: for albinism [28], and adult neurofibromatosis 1 [29]: development and validation of burden assessment tools. Health-related quality of life was explored in infants and children with interstitial lung disease [30].

Methodological aspects – Several tools for harmonizing our approaches in our own country and in Europe have been developed. We defined a way to federating the identities of RD patients [10]. Patient information in RD registries is generally collected as de-identified data from numerous sources, requiring the data to be federated. Transforming nominative data into de-identified data is thus a key issue, while minimizing the number of identity duplicates. We proposed a method enabling patient identity federation and RD data de-identification while preserving the pertinence of the provided data with a RD patient identifier.

A critical issue for cohort information platforms is to enforce strict control on access privileges and particularly for patients themselves. Cerberus, a comprehensive access control Scheme was designed to provide an access control scheme that covers design, implementation, deployment and maintenance operations [31]. It enables a targeted access to the RaDiCo GENIDA information platform by patients themselves who can capture their own data, or via an access to targeted medical data through explicit authorization by the study sponsor, when required, to practitioners and/or researchers.

Together with other European colleagues, we proposed an overview of the current situation and experiences of the national RD registries in Europe [32] and suggested recommendations for improving the quality of RD registries [33, 34]. These two papers illustrated part of the design of our e-Health approach. Moreover, RaDiCo is involved in the French PNMR3, notably in its 11.4 Action that reinforces the role of RaDiCo related to the integration of RD research data while developing operational links with the national RD databank by offering technical and ethical-regulatory support for the setting up by the RD health networks of new interoperable data warehouses and by studying the possibility of accommodating them on the RaDiCo platform. We also established operational links with the data platform of the European Joint Program on RD [35] and the ERNs currently being set up.

## Discussion

The RaDiCo program is intended to promote, through the support of RD e-cohorts’ projects, the collection of phenotypic data for epidemiological and clinical research purposes in connection with basic and translational research. To this end, we implemented an operational team (cf. Material and Methods section) with the mission to establish a centralized platform of expert services and tools to ensure installation and follow-up of the RD e-cohorts, via consulting services, for instance legal & regulatory services, or clinical research quality tools, development and provision of innovative information technology tools (electronic case report forms (eCRF), interoperability solutions, or access to e-health tools).

The preparatory period of the cohorts was slowed down by the uncontrollable delays required to obtain all the ethical and regulatory authorizations, as well as the evolving regulations both at the EU level, with the new European regulation on personal data safety and security (GDPR) [36], and at the national level, with modifications of the law on research involving human subjects (French «Jardé law») [37]. Moreover, a consortium agreement had to be signed between the partners for formalizing legal links between them, setting the modes of governance of the project on scientific, strategic and operational plans, and outlining the scientific valorization in terms of intellectual property, publications and citations of the project. A set of 19 Key Performance Indicators (KPIs) was implemented to enforce the cohorts’ follow-up (Table 4). Once initiated, the 13 ongoing RD cohorts progressed appropriately and the first results related to the secondary objectives of several cohorts have been published in international peer-reviewed journals.

**Table 4:** RaDiCo e-cohorts key performance indicators (KPI)

KPI 1	Methodology/biostatistics expertise as SC member
KPI 2	One Scientific Committee meeting per year with reporting
KPI 3	One yearly global RaDiCo progress report
KPI 4	Yearly cohort-specific progress reports
KPI 5	Reinforcement of the biostatistical expertise
KPI 6	Number of Scientific/Steering Committee meeting repoarts according to the predefined scientific agenda of each cohort (protocol and timelines)
KPI 7	Cohorts’ progress reports including accrual rate of prevalent and incident cases recruited
KPI 8	Number of recruitment sites in France/Europe per cohort
KPI 9	Number of per site actual recruitments per cohort, according to pre-defined six-month objectives
KPI 10	Loss to follow-up of included patients/death
KPI 11	Completeness of data collection
KPI 12	Per cohort data management status
KPI 13	Whenever relevant, number of bio samples collected/associated to clinical data collected
KPI 14	Integration to the National RD Plan (PNMR3), RD European Joint Program (EJP) and European Reference Networks activities
KPI 15	Scientific publications/communications produced by each cohort and the RaDiCo platform
KPI 16	Number of Specific Research Projects exploiting each cohort’s resources (data and biocollections)
KPI 17	Number of EU projects (H2020/COST/ERN) valorising/integrating RaDiCo cohorts
KPI 18	Implementation and follow-up of the Infrastructure as a Service, also proposed as a service to other cohorts of non-rare diseases; conformity to the European General Data Protection Regulation through yearly security audit results
KPI 19	Building an exchange framework with the French Health Data Hub Services (https://www.health-data-hub.fr/)

The costs and management of informatics providers and Clinical Research Organizations are important. Public–Private Partnerships helped to build our sustainability plan. Legal contracts were set up for consortium and collaboration agreements, enabling data collection and sharing.

At the cohort level, we industrialized our production processes, as well as tooling methods such as standardized operating procedures or documents shared online. Recurring difficulties are the shortage of manpower for data entry, the difficulties in identifying cases in the different hospital information systems, or the legal barriers to collect data from deceased children. We implemented solutions through identifying key data and prioritization of the fields related to the main objectives to be completed by the clinical research technicians with support from residents for filling the medical information. Clinical research technicians have been recruited by the RDHNs and specific resources have been obtained through targeted research projects supported by Public–Private Partnerships or applications to specific grants.

We explored the impact of RaDiCo for the medical community. Based on current information provided by all the cohorts’ participating teams, the program should contribute to significant improvements in patients’ care and outcome. Indeed, the design of the cohorts is made in such a way that it allows structured collection of disease symptoms at various stages of the pathological processes. Expected benefits include better knowledge of the natural history of all the investigated RDs, recognition of relevant comorbidities, novel proposals for healthcare management, and, ultimately, better quality of life for the patients and their families. In line with the objectives specifically defined for each cohort, other expectations are: identification of relevant disease biomarkers for diagnosis, disease severity and exacerbations; patient selection for clinical and therapeutic trials; and production of novel quality of life questionnaires. The development of electronic health tools is also among the secondary objectives of several cohorts. These tools are designed to help patients to self-report their symptoms as well as their medical management, and ultimately to assist the health care providers to make appropriate changes to medication use.

Altogether, the cohort studies should help progressing towards a more personalized medicine, particularly for appropriate investigations and therapeutic strategies. They should contribute to decrease the burden of these chronic diseases and to improve their socio-economic impact.

The RD cohorts are multidisciplinary and, for most of them, include molecular diagnostics and research laboratories. Organization and standardization of biological sample collections (biobanks) are part of the program. Strong interactions and collaborations between clinicians and basic scientists are developed to identify genetic and environmental determinants in well-defined groups of patients, to establish genotype/phenotype correlations, and to progress in the understanding of the underlying molecular and cellular mechanisms.

Rare diseases carry high morbidity and mortality; they represent a significant burden for the health care systems. Evaluation of the economic impact of RDs is therefore critical. This implicates a dedicated focus on the cost-effectiveness analyses, which evaluate both the costs and results of the health care systems and organizations applied in RDs. The final goal is to appropriately allocate the health care resources to the RD patients all over the country. This implies that RD are adequately traceable in the national health information systems. In line with these needs, the Ministry of Health has set up the RD national data bank. All the reference centers and their network of teams in France have the obligation to implement this data bank. Standardized and detailed patient information collected through the cohort programs support the production of economic indicators. Moreover, access to the French Health Data Hub [38] that is dedicated to cross-referencing the health databases and facilitating their use by research teams with full respect of user privacy of the health system, will open access to patients’ health expenditures from three main databases: the National Health Insurance Fund [39], hospital stays from the French hospital discharge database (PMSI) [40] and to the causes of death from the Inserm Centre for Epidemiology on the Medical Causes of Death, in charge of the national statistics of medical causes of death [41]. Economic indicators aim at monitoring the development and implementation of national strategies for RDs, which imply homogeneity and standardization of patient management by the medical structures dedicated to RDs.

All the teams of the RaDiCo cohorts are members of the RD Coordinating Centers in France. Among their tasks, through their organizations and their multidisciplinary teams, all the cohorts actively participate in the production of the RDs National Protocols for Diagnosis and Care [42].

The ERNs are networks of RD centers of expertise, healthcare providers and laboratories that are organized across borders. Most of the medical teams associated with the RaDiCo program are involved in the activities of the ERN in their field. They participate in the implementation of European data registries, in the production of clinical practice recommendations, and in the setting of epidemiological and economic indicators.

## Conclusion

The RaDiCo program promotes the collection of RD phenotypic data of different types for epidemiological and clinical research purposes in connection with basic and translational research. The RaDiCo platform offers the cohorts an information system based on the cloud principle (Information as a Service) together with a common core of services and specific procedures for some cohorts to ensure installation and follow-up of the RD e-cohorts. The information system of RaDiCo can drive a virtually unlimited number of RD e-cohorts, a major strength compared to other IS that usually deal with only one cohort. Currently, 13 RD e-cohorts are implemented and other national, European and international cohorts are targeted.

## Material and methods

### RaDiCo’s national call for implementing RD cohorts

In 2014, RaDiCo launched a national call for RD Cohort proposals organized as a two-stage procedure (letter of intention/full application). Guidelines and templates for candidate cohorts were designed and made accessible on line to applicants. The full dossiers were evaluated by independent international experts (at least 3 per project), with pre-established evaluation criteria.

### RaDiCo’s governance

An Institutional Committee is composed of the founding institutions including the following: Inserm (coordinating institution), 6 Universities: Paris Descartes and Paris Diderot now merged in the University of Paris, as well as Sorbonne, Paris-Est Créteil, Aix-Marseille and Montpellier universities; and the « Entreprises du Médicament», together with the Alliance for Research and Innovation of the Industries of Health. These bodies are linked to the RaDiCo program by an agreement established with the National Research Agency.

Among other missions, the Institutional Committee regularly follows the implementation of the project including the budget implementation. It validates, on proposal of the Scientific Committee, the annual program of activities. It decides the creation, composition, missions and the functioning of all *ad-hoc* governing bodies.

The RaDiCo Scientific Committee for the whole Program is composed of 18 members with complementary expertise associating coordinators of RD Coordinating Centers, RD Healthcare Networks, epidemiologists, biostatisticians, experts in Information Systems, molecular geneticists, directors of Inserm Research Units in the field of RDs, and representatives of Inserm Thematic Institutes. Its role is to support the Executive Committee concerning strategic issues and scientific governance of the program, including management of the call for projects and specific follow-up of cohorts; and to contribute to the national call for RD cohort projects launched by RaDiCo in 2014.

An Executive Committee is shared by all the cohorts. It is composed of 4 members with complementary expertise (SG, AC, PL and SA). Its role is to ensure the implementation, development and monitoring of the entire program. Its main missions are operational management and deployment planning of the sustainability and internationalization of the RaDiCo platform, the links with each RD cohort, and factorization of the shares. It is responsible for the scientific management of the Program which is implemented at different levels: by the Executive Committee, the Scientific Committee, the Inserm Thematic Institutes of “Public health”, “Technology for Health”, “Genetics, Genomics and Bioinformatics”, as well as by the 3 scientific governance bodies of each RD cohort. Indeed, each cohort has its own governance, on a scheme comparable to that of the whole program, with a Plenary Committee, a Scientific Committee and an Executive Committee.

The RaDiCo team is directed by a Scientific & Operational Director who leads 24 employees in close collaboration with the Executive Committee. The team acts within 3 different sub-units: Clinical research, Biometry, and Information System & e-Health.

### The RaDiCo platform

The RaDiCo platform is managed by the IS team together with the e-Health team. It uses exchange format and data security in compliance with the European directive on the General Data Protection Regulation. The RaDiCo work plan for each cohort is structured to achieve four levels of interoperability: technological, semantic, syntactic, and institutional. This allows networking and optimising the use of existing RD patient cohorts at the EU level, while allowing integration of new types of data and technologies. The data of each RaDiCo cohort and associated services have to be compliant with the FAIR principles (Findable, Accessible, Interoperable and Reusable) (https://www.go-fair.org/fair-principles/) by both people and computers. An important step in the FAIR data approach is to publish existing and new datasets from RaDiCo cohorts in a semantically interoperable format that can be understood by computer systems.

### The RaDiCo clinical research unit

RaDiCo Clinical research unit helps the PIs of the cohorts to finalize their project according to the SPIRIT recommendations (https://www.equator-network.org/reporting-guidelines/spirit-2013-statement-defining-standard-protocol-items-for-clinical-trials/), to submit the protocol to the ethical and legal authorities and obtain the corresponding agreements; design the e-CRF (electronic Case Report Form); implement the e-CRF on the IS platform; organize the data capture and the data quality control; monitor the follow-up according to 19 Key Performance Indicators; perform the data management; data freeze the data; prepare and perform the data analysis.

Patients are recruited through RD clinical sites for 12 cohorts. One cohort (GENIDA) proposes self-enrollment and self-reporting according to a pre-defined chart. Collected data for a given cohort were defined in an e-CRF. They are collected along with time (according to each cohort design, either quarterly, semi-annual or annual) and managed via the RaDiCo platform through REDCap (Research Electronic Data Capture) which is a secure, web-based application from the Vanderbilt University, USA. It was designed to support data capture for research studies, providing an intuitive interface for validated data entry; audit trails for tracking data manipulation and export procedures; automated export procedures for seamless data downloads to common statistical packages; and procedures for importing data from external sources.

## Data Availability

The data of the cohorts are not available presently since they are still running and primary objectives have not been analyzed yet. The datasets analyzed will be available from the PIs of the corresponding cohorts on reasonable request.
